# Diagnostic Accuracy of Combination of Multiparametric MRI PI-RADS Score v2.1 and Prostate-Specific Antigen Density for Prostate Cancer Detection

**DOI:** 10.7759/cureus.80238

**Published:** 2025-03-07

**Authors:** Ashrita Shetty, Jahnavi Gadupati, Bhagyalakshmi Bommineni, Sowmya Chikatla, Umesh Krishnamurthy, Ramesh D

**Affiliations:** 1 Radiology, M. S. Ramaiah Medical College, Bengaluru, IND; 2 Urology, M. S. Ramaiah Medical College, Bengaluru, IND

**Keywords:** lesion volume, multiparameteric mri, pirads v2.1, prostate cancer, psa density

## Abstract

Introduction

Prostate cancer (PCa) is the second most commonly diagnosed cancer in men worldwide. The Prostate Imaging-Reporting and Data System version 2.1 (PI-RADS v2.1) scoring system using multiparametric magnetic resonance imaging (mp-MRI) increases the accuracy for the assessment of clinically significant PCa. This study evaluates the diagnostic accuracy of a combination of PI-RADS v2.1 scores with prostate-specific antigen density (PSAD) for the detection of PCa, using biopsy outcomes as the gold standard, as well as the diagnostic accuracy of the combination of PI-RADS 3 lesion volume and PSAD.

Methods

This is single-center cross-sectional retrospective study including 54 subjects with serum PSA values > 4 ng/mL, who were referred for prostate mp-MRI. All patients underwent subsequent transrectal ultrasound (TRUS)-guided biopsy. Data collected includes PSA value, mp-MRI characteristics of the lesion, and histopathological findings. PI-RADS v2.1 score and PSAD were used to evaluate the diagnostic accuracy of this combination.

Results

In our study, the optimal PSAD cutoff was >0.18 with an area under the curve (AUC) of 0.897, indicating good diagnostic performance. The combination of PI-RADS v2.1 score ≥ 3 and PSAD ≥0.18 increased diagnostic accuracy, with a sensitivity of 96.97% and specificity of 71.43%. However, lesion volume was not a significant predictor of PCa.

Conclusion

In summary, our study demonstrates that the combination of PI-RADS score and PSAD yields higher diagnostic accuracy for the detection of PCa (p < 0.001) than using the PI-RADS score alone. We found an optimal PSAD cutoff of 0.18, which differs from the international consensus of 0.15. However, it cannot be used as a substitute for definitive pathological diagnosis but can be used in combination for better risk stratification, counselling, and management of patients with elevated PSA levels. Combining PI-RADS 3 lesion volume and PSAD did not have statistically significant results in our study.

## Introduction

Prostate cancer (PCa) is currently the second most commonly diagnosed cancer in men worldwide [1]. The increased burden of PCa is expected worldwide, with an estimated 1.7 million new cases and 499,000 new deaths by the year 2030 [2]. PCa is seen in men above 65 years of age [1]. Prostate-specific antigen (PSA) is a common screening tool, and subsequent transrectal ultrasound (TRUS)-guided biopsy has significantly increased detection of PCa. However, this also leads to increased diagnosis of clinically not significant PCas and the risks of overdiagnosis and overtreatment [3].

For early diagnosis of PCa, multiparametric magnetic resonance imaging (mp-MRI) is an important imaging approach for local staging and assessing the aggressiveness of cancer. Currently, mp-MRI is considered the best imaging approach for the detection of PCa. However, its unavailability across all hospitals, higher cost of imaging, and shortage of experienced radiologists are a few of its limitations [3-5]. Prostate Imaging-Reporting and Data System version 2 (PI-RADS v2) recommends T1- and T2-weighted imaging (T2-WI), diffusion-weighted imaging (DWI), and dynamic contrast-enhanced (DCE) sequences as part of mp-MRI for a detailed evaluation and characterization of lesions [6].

PI-RADS v2 is a widely accepted scoring system worldwide to assign an overall score (1-5) for a lesion, with PI-RADS 1 indicating a low probability of significant disease and PI-RADS 5 indicating a high probability of significant disease. Although PI-RADS v2 has been used broadly worldwide, it has limitations in terms of inter-reader reproducibility. PI-RADS v2.1 recommends minor adjustments aimed to simplify assessment and reduce inter-reader variability while maintaining the overall scope and principle of version 2. These adjustments are intended to enhance consistency in interpretation of mp-MRI findings for PCa diagnosis [7,8].

This combination of targeted biopsies and mp-MRI imaging in biopsy-naïve patients increases the diagnosis rates of PCa [9,10]. To improve the diagnostic accuracy of PCa in patients with PSA above 4 ng/mL, a new entity is being used, PSA density (PSAD) (total serum PSA / prostate volume). This helps to determine the requirement for further evaluation with biopsy [11,12]. PI-RADS 3 lesions are equivocal and can be grouped into two risk categories based on lesion volume and PSAD category to determine the necessity of biopsies in such lesions [13,14].

The objective of current study is to determine diagnostic accuracy of the combination of PI-RADS v2.1 score and PSAD for the detection of PCa, with biopsy being the gold standard test. The study also aims to determine diagnostic accuracy of PSAD and lesion volume in PI-RADS 3 lesions.

## Materials and methods

Ethical Committee approved this cross-sectional single institution retrospective study and written consent was obtained from all 54 subjects. Subjects with PSA value greater than 4 ng/mL, referred from the department of urology for mp-MRI from May 2023 to May 2024 were enrolled in the study. These patients underwent subsequent TRUS-guided biopsy. Histopathological assessment of PI-RADS 2 lesions was done post-TURP.

Inclusion criteria

Patients who underwent mp-MRI for prostate evaluation and subsequent histopathological assessment at our institute were included in the study.

Exclusion criteria

The study excluded patients with prior prostate biopsy (within six weeks), those with prior prostate surgery, and those who had incomplete or non‑diagnostic MRI. Men without focal abnormalities on mp-MRI with PI-RADS score 1 were excluded from this study.

The study collected the following data: age, family history of PCa, baseline PSA value, history of previous biopsy, mp-MRI prostate volume, lesion mp-MRI characteristics (whether they were located in the peripheral or transition zone, PI-RADS v2.1 score, three-plane diameters, lesion volume), and lesion Gleason scores.

MRI was performed on 1.5T/ 3T MRI systems (Siemens MAGNETOM Avanto 1.5T System with 8-array body coil and Siemens MAGNETOM Vida 3T System with 18-array body coil, Siemens, Munich, Germany) using phased abdominal array coil. The routine protocol includes T2-WI, DWI, and corresponding apparent diffusion coefficient (ADC) maps, and DCE sequences in the axial planes covering the prostate and seminal vesicles. DWI sequence was separately acquired at higher b-values of B 600 and B 1800. This technique improves tumour conspicuity by enhancing the contrast between cancerous and healthy tissue. For the DCE images, an intravenous injection of gadolinium chelate at a dose of 0.2 mL/kg was given at a rate of 2.5 mL/s.

Interpretation was performed by two independent radiologists (with 10 and 6 years of experience, respectively, in prostate mp-MRI). The readers were blinded to the previous MRI reports and pathological findings. The readers were aware of each patient’s clinical findings during the PI‑RADS scoring, which was based on PI‑RADS v2.1. The prostate gland volume is calculated as follows: (maximum anteroposterior dimension) × (maximum longitudinal dimension) [in the midsagittal T2-WI] × (maximum transverse dimension) [in the axial T2-WI] × 0.52.

The PSAD was calculated as follows: PSAD = PSA / prostate volume, reported in ng/mL/cc [15]. PI‑RADS v2.1 suggests the probability of the presence of clinically significant PCa (csPCA) utilizing a 5‑point scoring scale.

csPCa is defined on the basis of Gleason score ≥ 7 (including 3 + 4 with a prominent but not predominant 4 component) at histopathological examination, or volume >0.5 cm3, or extraprostatic extension [15].

In patients with visible lesions on MRI, four-core biopsy per target was initially performed, followed by a 12-core biopsy. Targeted biopsy was performed using the BK 3000 ultrasound system. TRUS-guided prostate biopsy was performed using 18G × 25 cm automated gun by the Department of Urology. The standardized 12‑core TRUS‑guided systematic biopsy (six in the peripheral zone and six in the transitional zone) was performed. Pathologic assessment was done by experienced pathologists of our hospital in accordance with the 2019 International Society of Urological Pathology (ISUP) Consensus Conference [16].

Data analysis was conducted using SPSS 22 Version software (IBMCorp., Armonk, NY, USA). Categorical data were presented in the form of frequencies and proportions. The chi-square test was used to test significance for qualitative data. Continuous data were presented as mean and standard deviation. Normality of the continuous data was tested using the Kolmogorov-Smirnov test and the Shapiro-Wilk test. Validity of the screening test was plotted by receiver operating characteristic (ROC) curve, showing the best cutoff values for sensitivity and specificity of the study.

## Results

The study included 54 participants, with a mean age of 71.35 ± 8.265 years (range: 51-83 years). The mean PSA value in our participants was 29.63 ± 80.16, mean prostate volume was 49.67 ± 31.56, mean PSAD was 0.50 ± 0.37, and mean lesion volume was 0.60 ± 0.28 (Table 1). The mean PSA levels in our participants showed higher heterogeneity due to two cases of prostatitis that mimicked PI-RADS 5 lesions and two cases of locally advanced cancers with a PSA value of >100 ng/mL. PSAD was higher in malignant lesions than the benign group (median PSAD was 0.41 ng/mL/cc and 0.17 ng/mL/cc for malignant patients and benign patients, respectively; P < 0.01) (Table 1).

**Table 1:** Distribution of age, PSA, prostate volume, PSAD, and lesion volume PSA, prostate-specific antigen; PSAD, prostate-specific antigen density

	Mean	SD
Age	71.35	8.265
PSA value	29.63	80.16
Prostate volume	49.67	31.56
PSAD	0.50	0.37
Lesion volume	0.60	0.28

**Figure 1:**
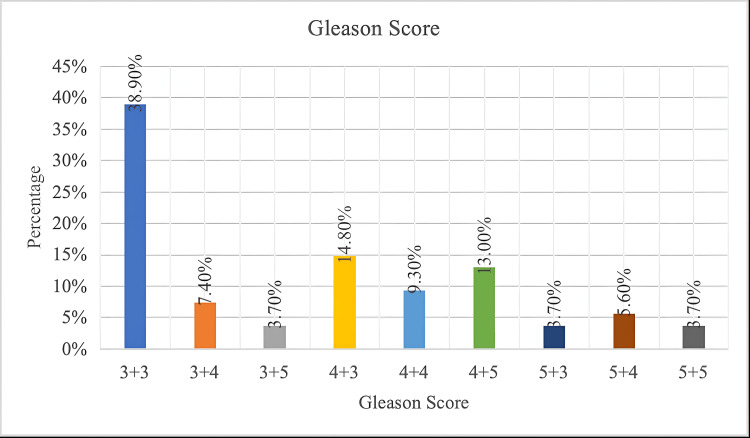
Bar diagram showing Gleason score distribution In the study, majority of subjects had a Gleason score of 3+3 (38.9%), followed by 4+3 (14.8%) and others.

Location of lesions in 54 patients were assessed using mp-MRI. In our study, 42.6% of lesions were located in the peripheral zone (23 subjects), 40.7% lesions in the transition zone (22 subjects), and 16.7% of lesions in both the transition and peripheral zones (9 subjects). In the study, majority of subjects had a Gleason score of 3+3 (38.9%), followed by 4+3 (14.8%) and others (Figure 1).

**Figure 2:**
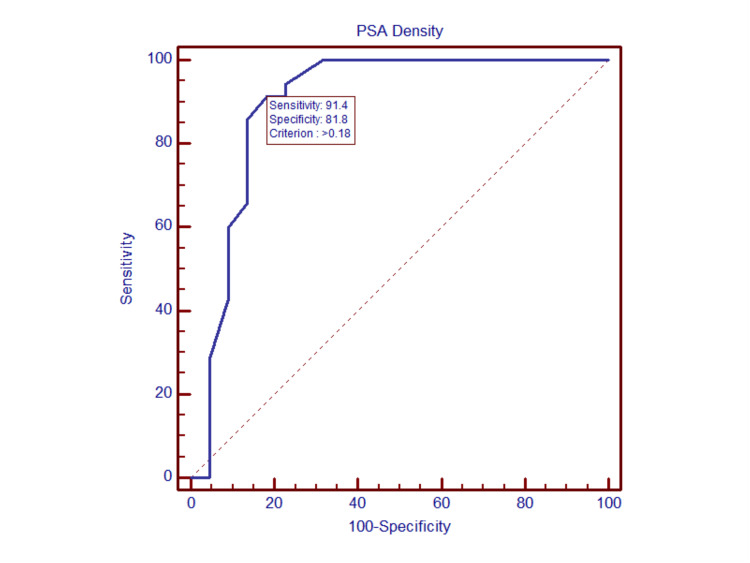
ROC curve showing PSA density in predicting prostate malignancy The ROC curve analysis for predicting prostate cancer showed that the AUC value of PSA density was 0.897 (95% CI: 0.787–0.962; p = 0.0001), suggesting that PSA density is a good marker for the diagnosis of prostate cancer. The ideal cutoff as per Youden’s index method was found to be >0.18, with a sensitivity of 91.4% and specificity of 81.8% in predicting prostate cancer. PSA, prostate-specific antigen; ROC, receiver operating characteristic

The area under the curve (AUC) for PSAD in the prediction of PCa by ROC curve analysis showed a value of 0.897 (95% CI: 0.787-0.962; p = 0.0001), suggesting that PSAD is a good marker for diagnosis of PCa. The ideal cutoff as per Youden’s index method was found to be >0.18, with a sensitivity of 91.4% and specificity of 81.8% in predicting PCa (Figure 2).

PSAD was grouped into three categories: < 0.15, 0.15-0.30, and > 0.30. In this study, 33 cases were malignant on histopathological analysis, and PSAD cutoff among these lesions resulted in the detection of 87.9% malignant and 12.1% of benign lesions. The other 21 cases were benign on histopathological analysis, and PSAD cutoff among these lesions resulted in the detection of 66.7% benign and 33.3% malignant lesions. There was significant association between biopsy findings and PSAD. In this study, PSAD of ≥ 0.15 had a sensitivity, specificity, positive predictive value (PPV), and negative predictive value (NPV) for the detection of csPCa of 91.4%, 81.8%, 80.56%, and 77.78%, respectively.

In this study, a PI-RADS v2.1 score of ≥3 had a sensitivity, specificity, PPV, and NPV for the detection of csPCa of 92.91%, 71.43%, 83.33%, and 83.33%, respectively.

**Figure 3:**
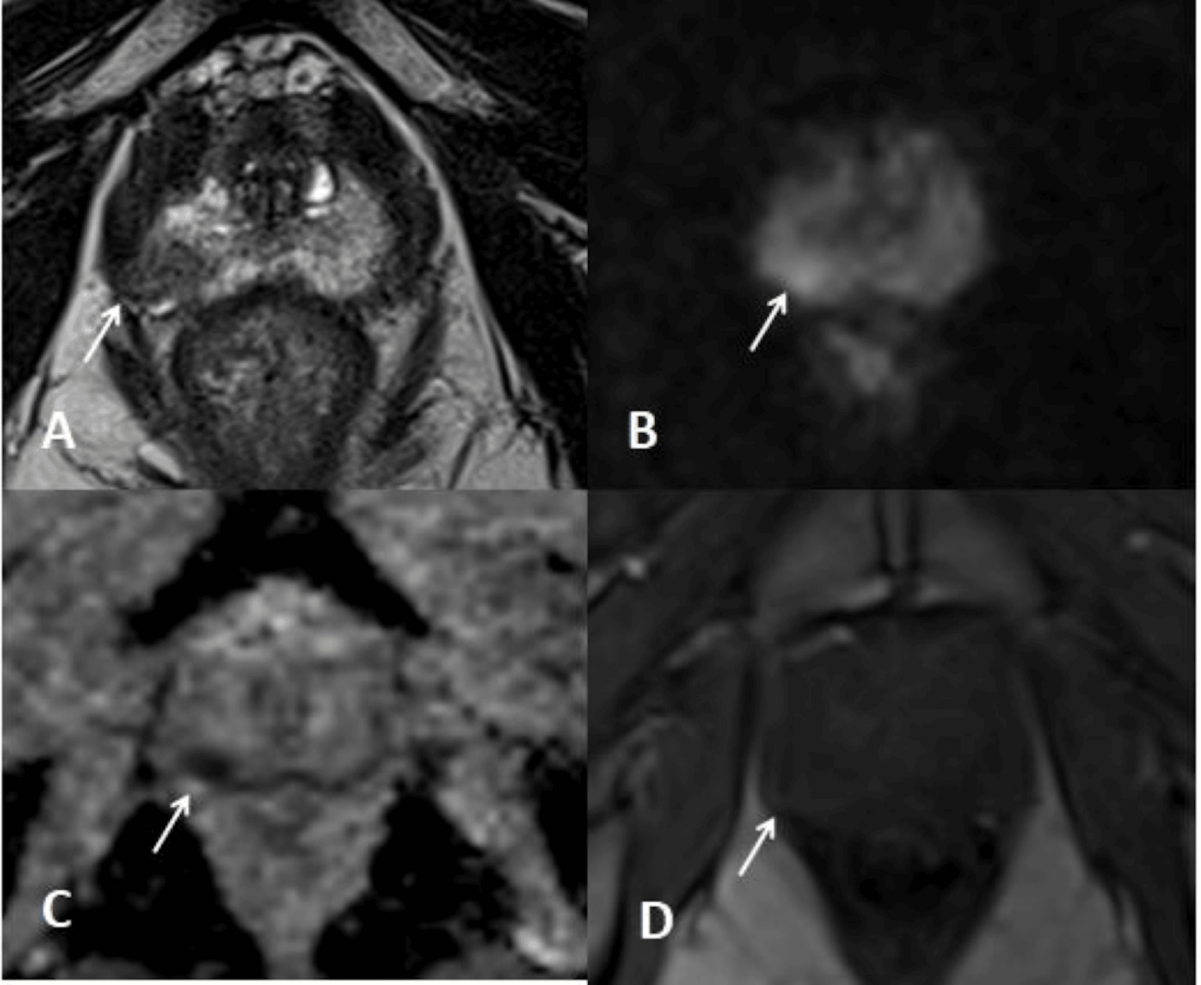
Reference case A 63-year-old man presented with complaints of urinary incontinence. PSA was elevated at 5.9 ng/mL. On ultrasound examination, there was grade 1 prostatomegaly with features of cystitis. PSA density of 0.19 ng/mL/cc. MRI of the prostate was performed for further evaluation. T2-weighted axial section imaging (Figure 3A) showed an ill-defined hypointense area in the right posterior-lateral peripheral zone with hyperintensity on DWI (Figure 3B) and markedly low values on ADC (700-800x 10-6 mm^2^/s) (Figure 3C), without early or contemporaneous post-contrast enhancement, suggestive of a PI-RADS 3 lesion (Figure 3D). The lesion on histopathological examination was diagnosed as an adenocarcinoma. ADC, apparent diffusion coefficient; DWI, diffusion-weighted imaging; PI-RADS, Prostate Imaging-Reporting and Data System; PSA, prostate-specific antigen

**Figure 4:**
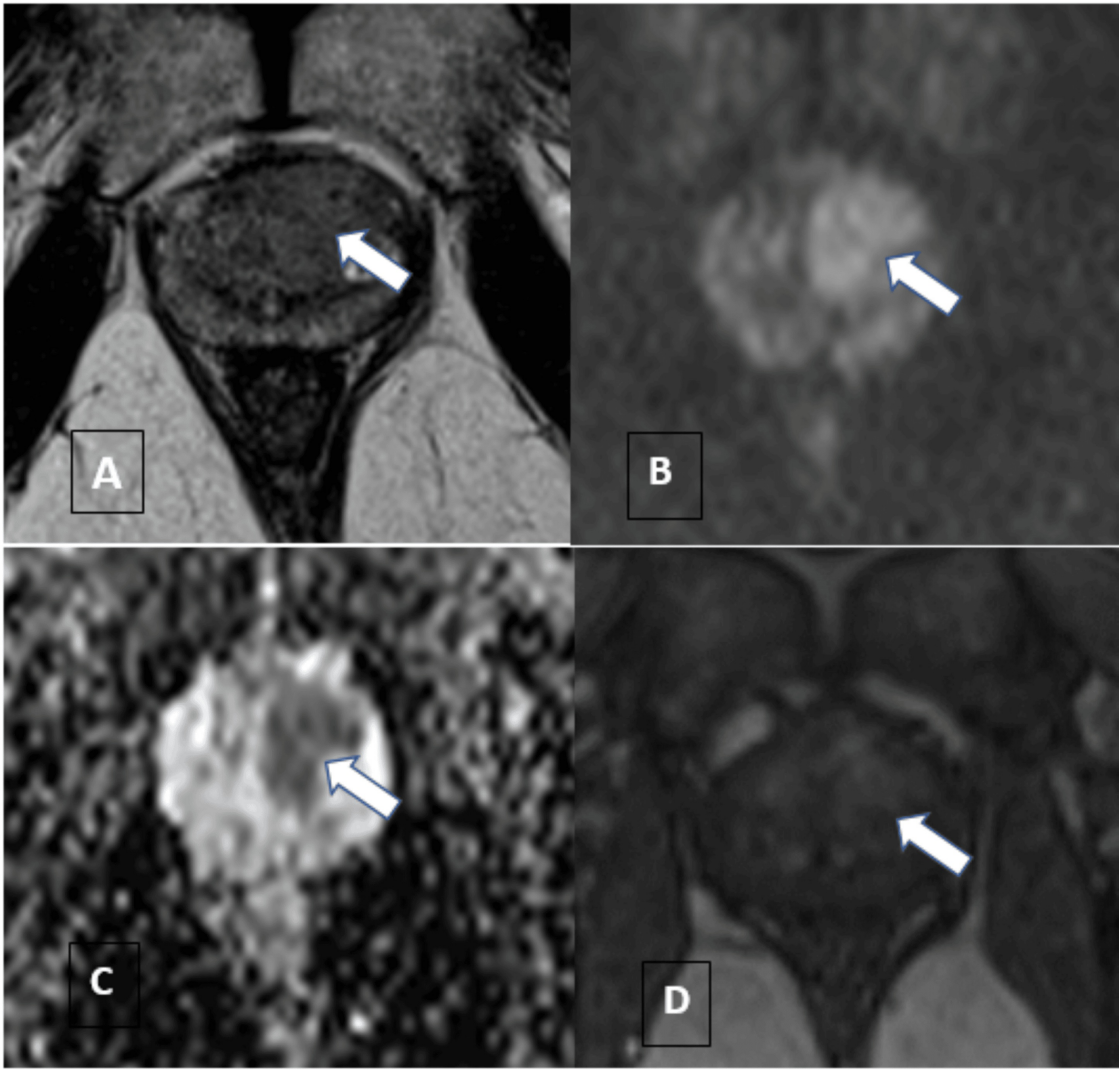
Reference case A 52-year-old man presented to the urology clinic with complaints of urinary incontinence. The serum analysis showed an elevated PSA of 7.6 ng/mL. Ultrasound examination demonstrated features of chronic cystitis with prostatomegaly.  Mp-MRI showed an enlarged prostate with a volume of 32 cc and PSA density of 0.23 ng/mL/cc. On T2-weighted axial section imaging (Figure 4A),  there was a lenticular area of hypointensity in the left anterior transition zone with hyperintensity on DWI (Figure 4B) and low values on ADC ( Figure 4C). DCE images demonstrated an early heterogenous enhancement within the lesion (Figure 4D), suggestive of a PI-RADS 4 lesion.  The lesion on histopathological examination was diagnosed as an adenocarcinoma. ADC, apparent diffusion coefficient; DCE, dynamic contrast-enhanced; DWI, diffusion-weighted imaging; PI-RADS, Prostate Imaging-Reporting and Data System; PSA, prostate-specific antigen

Combination of PI-RADS v2.1 score of ≥3 and all PSAD categories yielded the highest number of overall csPCa detection. There was significant association between biopsy outcome and the combination of PI-RADS and PSAD findings in the detection of PCas. The sensitivity, specificity, PPV, and NPV for the detection of csPCa were 96.97%, 71.43%, 84.21%, and 93.75%, respectively (reference cases Figure 3 and Figure 4).

Lesion volume

Additionally, we also assessed the diagnostic accuracy of PI-RADS 3 lesion volume with PSAD. The ROC curve analysis for predicting csPCa showed that the AUC values of PI-RADS 3 lesion volume was 0.524 (95% CI: 0.286-0.753; p = 0.883), which was statistically insignificant and suggested that the lesion volume cutoff for PI-RADS 3 lesion cannot be used as a marker for the diagnosis of PCa.

## Discussion

In this study, we assessed PI‑RADS v2.1 score and PSAD to test its diagnostic accuracy in the detection of PCa. The higher PI‑RADS score positively correlates with higher diagnostic yield of PCa detection. The highest diagnostic accuracy of csPCa was obtained with PI‑RADS 5 lesion.

PI‑RADS v2.1 is a good scoring tool for the detection of csPCa, even in isolation. In our study, a PI‑RADS score of ≥3 yielded a sensitivity, specificity, PPV, and NPV of 92.91%, 71.43%, 83.33%, and 83.33%, respectively. PI-RADS score predicted most PCas accurately (except for three cases).

In a study by Hofbauer et al. [15] including 704 men, PI-RADSv2 score showed a significant association with PCa detection (p <0.001), csPCa (p <0.001), and ISUP grade (p <0.001). We obtained similar results with PI-RADS v2.1 category showing a significant association with PCa detection (p <0.001).

In a study by Hötker et al. [7] including 229 men with two diagnosticians with different experience levels observed, PI-RADS v2.1 scoring system showed similar diagnostic performance and inter-reader variability when compared with version 2.0. The new changes in version 2.1 seem only to make a difference in a very small number of subjects.

A PI-RADS cutoff of ≥3 had the greatest sensitivity, which, in turn, aids in higher detection of overall PCa. Other studies used radical prostatectomy/systematic biopsies as reference standard and showed a varied range of sensitivities and specificities [14,17-20].

In our study, 42.6% of lesions were located in the peripheral zone (23 subjects), 40.7% in the transition zone (22 subjects), and 16.7% in both the transition and peripheral zones (9 subjects). Moreover, transition zone tumors accounted for nearly 40% of cases, which is at the higher end of normogram, which are otherwise estimated to be approximately 30% [21]. The lower sample size in our study has led to this deviation from normogram.

There was variation in our study in terms of sensitivity and specificity, which can be due to different PSAD cutoffs, differences in 1.5T/3T MRI machines, MRI acquisition protocols, variability of PI‑RADS scoring in peripheral and transition zones, biopsy protocol, differences in patient characteristics, and radiologist experience with the PI‑RADS v2.1 scoring system. There can also be inter-observer variability in mp-MRI prostate reporting, which depends on working experience.

The mean PSA value in our participants was 29.63 ± 80.16, showing higher heterogeneity due to two cases of prostatitis that mimicked PI-RADS 5 lesions and two cases of locally advanced cancer with a PSA value of >100 ng/mL. Chronic bacterial prostatitis can occur due to undertreated acute prostatitis or recurrent infections by lower urinary tract obstruction. Acute prostatitis present with local and systemic inflammatory symptoms. However, chronic prostatitis is usually indolent, without systemic symptoms [22]. The prevalence of prostatitis in men of Indian subcontinent is approximately 12 % compared to 8% in the western population [23]. In developing countries like India, most cases go undiagnosed and underreported, with actual prevalence being higher than what has been stated in the literature.

There was a significant association of PSAD in the detection of clinically significant cancer (p < 0.001) with a sensitivity of 91.4% and specificity of 81.8%. In our study, the optimal PSAD cutoff value obtained was 0.18 ng/mL/cc, with AUC of 0.897 (95% CI: 0.787 to 0.962), and the sensitivity, specificity, PPV, and NPV for the detection of overall PCa were 91.4%, 81.8%, 80.56%, and 77.78%, respectively.

In study conducted by Catalona et al. [24], PSAD cutoff of >0.10ng/mL/cc and >0.15 ng/mL/cc resulted in the detection of 77% (729/947) and 49% (461/947) cases of clinically significant prostate cancer, respectively. In an another study conducted by Pellegrino et al. [12], higher PSAD cutoff (≥0.20) was found to improve the detection of high-grade PCa cases.

Other items such as the percentage of free PSA and PSA velocity can be used for early detection of PCa. These provide comparable results, suggesting that PSAD may be used for biopsy decisions in place of % free PSA and PSA velocity [24]. Prostate health index is considered superior to PSAD in early cancer detection, particularly for PI-RADS 3 lesions [25]. However, its availability is limited across many institutes in developing countries.

In this study, we determined the diagnostic accuracy of the combination of PI-RADS v2.1 score and PSAD, which has a sensitivity of 96.97% and specificity of 71.43% in predicting PCa with statistically significant correlation (p < 0.001).

In a study conducted by Yadav et al. [5] in Asian population of Indian subcontinent on 58 patients, it was concluded that a PI-RADS v2 score of ≥4 with any PSAD levels and PI-RADS v2 score of 3 with PSAD of ≥0.15 ng/mL/cc resulted in the highest overall PCa.

In another study conducted by Nordström et al. [26] including 5,291 patients, csPCa detection was better when PSAD was combined with PI-RADS and additional clinical information (AUC 0.75 vs. 0.73, p < 0.05).

Diagnostic accuracy of lesion volume cutoff for PI-RADS 3 lesions was statistically insignificant (p = 0.883) and, hence, could not predict biopsy outcome in PI-RADS 3 lesions in our study. In a study performed by Kortenbach et al. [13] including 141 subjects with PI-RADS 3 lesions, it was reported that cases with lesion volume  ≥0.5 mL are more likely to develop csPCa than a tumor volume of  <0.5 mL and therefore can avoid overtreatment. We did not obtain similar results, likely due to a small sample size.

Patients with PSAD <0.15 ng/mL/cc did not yield csPCa and hence can avoid biopsies and their related complications. Hence, we determined that the combination of these two parameters (PI-RADS + PSAD) improved the predictive performance with the highest sensitivity and specificity in the detection of PCas than isolated use of PI-RADS score or PSAD.

The study limitations include a smaller sample size, which may have led to relative variation in the PSAD cutoff compared to other studies. Since this was a single-center study, it needs to be extended to multiple centers for broader applicability. We included studies using both 1.5T and 3T MRI, which could have subjectively altered the PI-RADS scores of lesions. Less studies are available on the Indian population, and PSA values can vary depending on sample population’s geographical location, which could have influenced variation in our study results. Another limitation pertains to the lesser access and availability of MRI fusion biopsy procedures in numerous medical centers of developing countries like India. MRI fusion biopsy is superior to standard 12-core biopsy, and the same study can be extrapolated with MRI fusion biopsy for better lesion detection. Larger studies with higher sample sizes are required in the Indian population to determine an optimal PSAD cutoff. Even with these limitations, the study holds significance and may contribute valuable insights into patient counselling and management.

## Conclusions

In summary, our study demonstrates that the combination of PI-RADS score and PSAD yields higher diagnostic accuracy for the detection of PCa than using the PI-RADS score alone. We found an optimal PSAD cutoff of 0.18, which differs from the international consensus of 0.15. However, it cannot be used as a substitute for definitive pathological diagnosis but can be used in combination for better risk stratification, counselling, and management of patients with elevated PSA levels. Combining PI-RADS 3 lesion volume with PSAD did not have statistically significant results in our study.
